# Genomic organization, annotation, and ligand-receptor inferences of chicken chemokines and chemokine receptor genes based on comparative genomics

**DOI:** 10.1186/1471-2164-6-45

**Published:** 2005-03-24

**Authors:** Jixin Wang, David L Adelson, Ahmet Yilmaz, Sing-Hoi Sze, Yuan Jin, James J Zhu

**Affiliations:** 1 Department of Poultry Science, Texas A & M University, College Station, TX 77843, USA; 2 Department of Animal Science, Texas A & M University, College Station, TX 77843, USA; 3 Department of Computer Science, Texas A & M University, College Station, TX 77843, USA

## Abstract

**Background:**

Chemokines and their receptors play important roles in host defense, organogenesis, hematopoiesis, and neuronal communication. Forty-two chemokines and 19 cognate receptors have been found in the human genome. Prior to this report, only 11 chicken chemokines and 7 receptors had been reported. The objectives of this study were to systematically identify chicken chemokines and their cognate receptor genes in the chicken genome and to annotate these genes and ligand-receptor binding by a comparative genomics approach.

**Results:**

Twenty-three chemokine and 14 chemokine receptor genes were identified in the chicken genome. All of the chicken chemokines contained a conserved CC, CXC, CX_3_C, or XC motif, whereas all the chemokine receptors had seven conserved transmembrane helices, four extracellular domains with a conserved cysteine, and a conserved DRYLAIV sequence in the second intracellular domain. The number of coding exons in these genes and the syntenies are highly conserved between human, mouse, and chicken although the amino acid sequence homologies are generally low between mammalian and chicken chemokines. Chicken genes were named with the systematic nomenclature used in humans and mice based on phylogeny, synteny, and sequence homology.

**Conclusion:**

The independent nomenclature of chicken chemokines and chemokine receptors suggests that the chicken may have ligand-receptor pairings similar to mammals. All identified chicken chemokines and their cognate receptors were identified in the chicken genome except CCR9, whose ligand was not identified in this study. The organization of these genes suggests that there were a substantial number of these genes present before divergence between aves and mammals and more gene duplications of CC, CXC, CCR, and CXCR subfamilies in mammals than in aves after the divergence.

## Background

Chemokines are a family of small chemoattrative peptides that were originally recognized to be involved in host defense as regulators of leukocyte trafficking, but more recently have also been shown to have roles in organogenesis, hematopoiesis, and neuronal communication [1]. Their cognate receptors belong to the Class A subfamily of G-protein coupled receptor superfamily [2]. Chemokines are believed to have originated from gene duplications and these genes underwent selection during recent evolutionary time [3]. All chemokines have a characteristic cysteine motif. Similarly, chemokine receptors may also be derived from a common ancestor through gene duplications. All chemokine receptors share high homology with the prototypical family member, rhodopsin [2].

Chemokines are highly basic proteins, 70 to 125 amino acids long. Sequence identity among chemokines is usually low; however, all share a typical overall tertiary structure, which consists of at least four cysteines that form two disulfide bonds. Chemokines are divided into two major (CC and CXC) and two minor (XC and CX_3_C) subfamilies based on their four conserved cysteines. The first two cysteines in the two major subfamilies are either adjacent (CC) or separated by one amino acid (CXC). The first two cysteines in the CX_3_C chemokines are separated by three amino acids, whereas the XC chemokines contain only two of the cysteines [4]. The CC chemokines can be further divided into two subcategories, MCPs (monocyte chemoattractant proteins) and MIPs (macrophage inflammatory proteins) based on their structural similarities [5]. The members of these two CC subcategories specifically attract mononuclear cells but not neutrophils. The CXC chemokines can also be divided into two subfamilies, one with an ELR (a conserved Glu-Leu-Arg preceding the first cysteine) motif, which is angiogenic and attracts neutrophils, and the other without the ELR motif, whose members do not attract neutrophils [6].

Chemokine genes are characterized by their chromosomal locations and similar gene structure. Most human CC and CXC genes are organized in gene clusters in mammalian genomes, such as human Chromosomes 4 and 17, and mouse Chromosomes 5 and 11 [7]. The genes encoding the CC subfamily contain three exons, whereas the CXC chemokine genes contain four exons [8,9]. The XC subfamily of chemokines contains two members in human but only one in mouse. CX_3_CL1 is the only known member of the CX_3_C subfamily in human, mouse, rat, and monkey. There are extensive conserved syntenies in the chromosomal regions containing chemokine genes between human and mouse.

Unlike chemokines, chemokine receptors share a higher degree of sequence identity within a species and between species. These receptors have characteristic seven alpha-helix transmembrane domains with a length between 340–370 amino acids and have up to 80% amino acid identity [1]. They also share an acidic amino terminus, a conserved sequence in the second intracellular loop, and one cysteine in each extracellular domain [10]. Most receptors can bind several chemokines of a single class with high affinity [11]. Like chemokines, most chemokine receptors are also clustered in a few chromosomal regions, such as human Chromosomes 2 and 3 [2]. Most amino acid sequences of chemokine receptors are encoded in one exon.

At present, 42 chemokine genes have been identified in human (24 CC, 15 CXC, 1 CX_3_C, and 2 XC) and 36 (21 CC, 13 CXC, 1 CX_3_C, and 1 XC) in mouse, whereas there are 11 receptors for CCLs, 6 for CXCLs, 1 for CX_3_CL, and 1 for XCL in human and mouse. Only 11 chicken chemokines including 4 CXC, 6 CC, and 1 XC and seven chicken chemokine receptors including 2 CXCR and 5 CCR have been reported in the literature [12-23]. Chicken chemokines share low sequence identity with mammals [24]. Therefore, it was very difficult to assign chicken chemokines to a specific mammalian counterpart based on sequence data alone. Because of limited sequence similarity, most of the reported chicken chemokines were not named in accordance with the systematic nomenclature of mammalian chemokines. The newly available chicken draft genome sequence and a large number ESTs allow systematic identification and annotation of chicken chemokine and cognate receptor genes. The objectives of this study were to systematically identify chemokine and chemokine receptor genes in the chicken genome, to name these genes according to existing systematic nomenclature, and to make ligand-receptor binding inferences based on comparative sequence analysis. The systematic nomenclature for these chicken genes was based on the phylogenetic trees and syntenies of chicken, human, and mouse genes, and ligand-receptor binding inferences were according to [37] and [38].

## Results

### Chicken chemokines and chemokine receptors

In addition to the 11 previously reported, 12 new chicken chemokine were identified. These include 7 new CC chemokines named CCL1L1 (BX935885), CCL3L1 (CF258095), CCL/MCP-L2 (CK610423), CCL/MCP-L3 (CK610627), CCL17 (BI067703), CCL19 (BX929857), and CCL21 (CR522995), 4 new chicken CXC chemokines named CXCL13a (BX262175), CXCL13b (BX264625), and CXCL13c (CR352598), CXCL15 (BX929947), and 1 CX_3_CL1 chemokine (assembled from CR389767, BI066258, BM426140, and our sequence: AY730688). Eleven reported chicken genes were also named accordingly as CCL1L2 (L34552), CCL5 (ah294, AY037859), CCL4L1 (MIP-1β, AJ243034), CCL/MCP-L1 (ah221, AY037860/ BX933162), CCL16 (k203, Y18692), CCL20 (ah189, AY037861), CXCL8a (cCAF, M16199), CXCL8b (K60, Y14971), CXCL12 (SDF-1, BX936268), CXCL14 (JSC, AF285876), and XCL1 (lymphotactin, AF006742). In summary, there are 13 CCL, 8 CXCL, 1 CX_3_CL, and 1 XCL genes identified in the chicken genome. The information used for the nomenclature is shown in the comparative genomic maps and phylogenetic trees.

**Figure 1:**
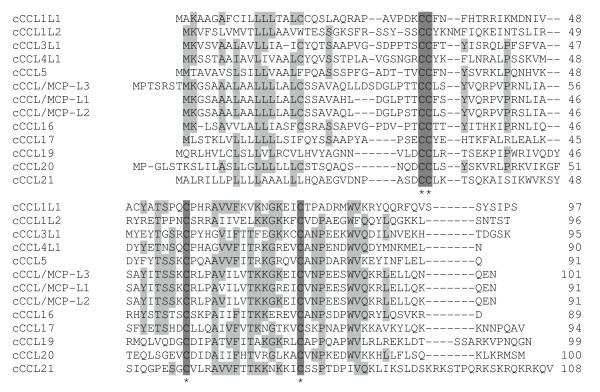
**Alignment of amino acid sequences of chicken chemokine CC subfamily**. Alignment gaps are indicated by dashes. Sequences with identical amino acid in at least 50% of chicken chemokines are highlighted in gray and conserved cysteine residues in dark gray.

**Figure 2:**
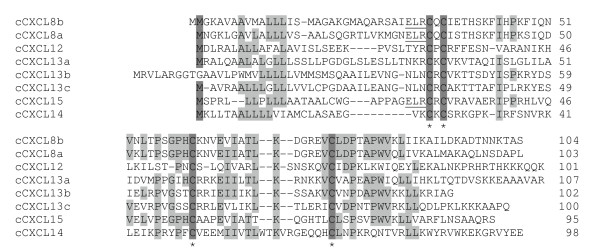
**Alignment of amino acid sequences of chicken chemokine CXC subfamily**. Alignment gaps are indicated by dashes. Sequences with identical amino acid in at least 50% of chicken chemokines are highlighted in gray and conserved cysteine residues in dark gray. The conserved ELR motifs are underlined.

**Figure 3:**
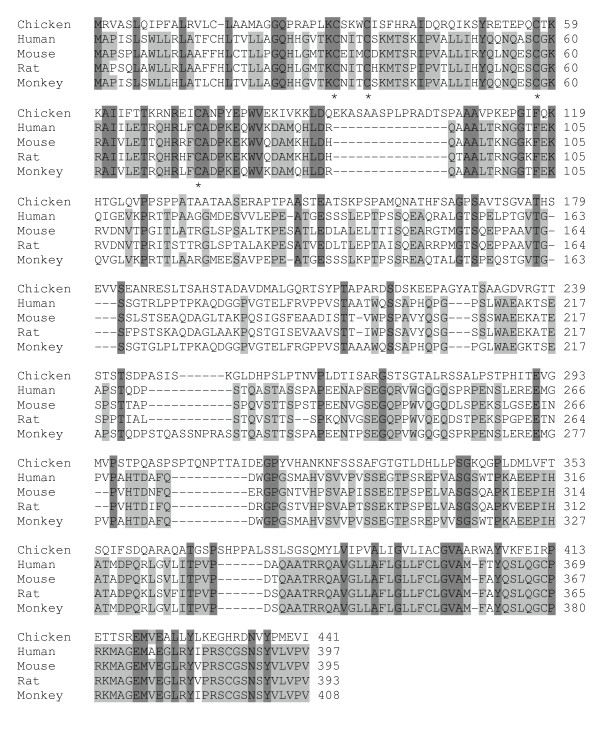
**Alignment of amino acid sequences of chicken, human, mouse, rat and monkey chemokine CX_3_CL1**. Alignment gaps are indicated by dashes. Sequences identical in all species are highlighted in gray. The asterisk represents the conserved cysteine residues.

Chicken chemokine amino acid sequence alignment shows that all chicken CC chemokines have four conserved cysteines with two adjacent cysteines at the N-terminus (Figure 1), whereas all chicken CXC chemokines have the conserved four cysteines with the first two cysteines separated by one amino acid (Figure 2). Both chicken CCLs and CXCLs show higher degrees of sequence similarity to each other in the signal peptide sequences and sequence regions containing the last two cysteines. Chicken CXCL8a, CXCL8b, and newly identified chemokine CXCL15 contain the ELR (Glu-Leu-Arg) motif. Only one chicken CX_3_C chemokine was found (Figure 3). The number of amino acid residues between conserved cysteines in all chemokines is highly conserved between chicken and human (Table 1).

**Table 1:** Chicken and corresponding human chemokine cysteine motifs

**Families**	**Chemokines**	**Chicken motif**	**Human motif**
CC	CCL1L1	CCX_25_CX_15_C	CCX_22_CX_15_C
	CCL1L2	CCX_24_CX_15_C	CCX_22_CX_15_C
	CCL3L1, CCL4L1, CCL5	CCX_22_CX_15_C	CCX_22_CX_15_C
	CCL16	CCX_22_CX_15_C	CCX_21_CX_15_C
	CCL17	CCX_22_CX_15_C	CCX_22_CX_15_C
	CCL19	CCX_25_CX_15_C	CCX_24_CX_15_C
	CCL20	CCX_24_CX_15_C	CCX_24_CX_15_C
	CCL21	CCX_25_CX_15_C	CCX_24_CX_17_C
	CCL/MCP-L1, -L2, -L3	CCX_22_CX_15_C	CCX_22_CX_15_C
CXC	CXCL8a, CXCL8b	CXCX_24_CX_15_C	CXCX_24_CX_15_C
	CXCL13a, CXCL13b, CXCL13c	CXCX_24_CX_15_C	CXCX_24_CX_15_C
	CXCL12	CXCX_22_CX_15_C	CXCX_22_CX_15_C
	CXCL14	CXCX_23_CX_19_C	CXCX_23_CX_20_C
	CXCL15	CXCX_24_CX_15_C	N/A^1^
XC	XCL1	CX_36_C	CX_36_C
CX_3_C	CX_3_CL1	CX_3_CX_21_CX_15_C	CX_3_CX_21_CX_15_C

^1 ^CXCL15 is not found in humans.

Chicken chemokines have limited amino acid sequence similarity compared to their human counterparts. Generally, chicken CXC chemokines share 27 to 60% amino acid identity with their human homologs except for CXCL12, which share 73% identity with human CXCL12. The length of chicken chemokine CXCL polypeptides ranges from 95 to 107 amino acids. Compared to their human homologs, chicken CXCL chemokine amino acid sequences are shorter except for chicken CXCL8a, CXCL8b, and CXCL12, which are 4, 5, 12 amino acids longer than their respective human homologs. In contrast, the sequence identities between human and chicken CCL chemokines are generally lower than those for CXCL chemokines, ranging from 25 to 56%. Chicken chemokine CCL polypeptides have 89 to 108 amino acids. Chicken CCL1L2, CCL5, and CCL17 have the same amino acid length as their human counterparts. Chicken CCL4L1 is shorter than corresponding human CCL4, whereas chicken CCL1L1, CCL3L1, CCL19, and CCL20 are longer than the corresponding human CCLs. These differences in length between human and chicken chemokines are mostly in the N- and C- termini.

Chicken CX_3_CL1 encodes 441 amino acids, longer than all mammalian CX_3_CL1 examined. It shares 20, 22, 20, and 22% amino acid identity with human, mouse, rat, and monkey CX_3_CL1, respectively. Sixty-six amino acid residues in chicken CX_3_CL1 are identical to residues in mammals, but the sequence identity between mammals is, as expected, much higher than that between chickens and mammals (Figure 3). There are more identical amino acids between chicken and mammals at both ends of the sequences.

Unlike the chemokines, all the chicken chemokine receptor genes were aligned with non-chicken chemokine receptor reference genes in the chicken genome browser. There was at least one chicken EST sequence aligned to each receptor gene except for CCR4. In addition to 7 reported receptors, 7 new chicken chemokine receptors were identified and named as CCR4 (predicted sequences: ENSGALT00000019505.1), CCR6 (CV039916, BU451770, and CK987456), CCR7 (predicted sequence: chr27_random_59.1), CCR8a (AJ720982), CXCR2 (BX258468), CXCR5 (AJ450829), CX3CR1 (CF252942, BU204148, and AJ443633). In contrast to chicken chemokines, chicken chemokine receptors share significant amino acid identity with their human receptor counterparts. The percents of amino acid identity between chicken and human chemokine receptors range from 48 to 81%. The lengths of these chicken receptors range from 335 to 382 amino acids. The complete sequence of chicken CXCR2 is unknown due to a sequence gap in the chicken genome sequence. The CXCR2 EST and a partial genome sequence contain the last 170 amino acids of the C-terminus.

**Figure 4:**
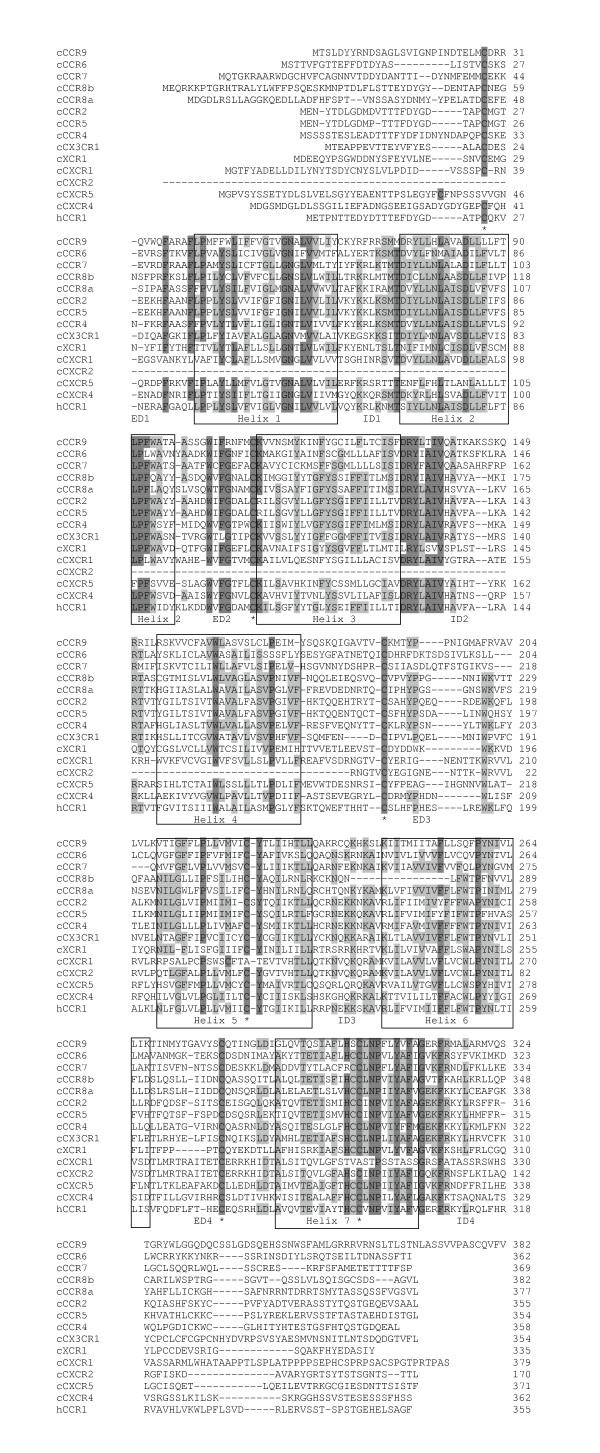
**Alignment of amino acid sequences of chicken chemokine receptors with human CCR1**. Alignment gaps are indicated by dashes. Sequences with identical amino acid in at least 50% or 85% of the chicken chemokines are highlighted in gray and dark gray, respectively. Asterisks represent the conserved cysteine residues. ED and ID denote extracellular and intracellular domains, respectively. Seven transmembrane spanning domains of chicken chemokine receptors were predicted using the SMART program and these consensus domains are indicated with a box. The N-terminal sequence of chicken CXCR2 is currently unknown.

Fourty-four amino acid residues were highly conservated (>85% homologies) among all chicken chemokine receptors (Figure 4). These receptors all have seven transmembrane helices and three extracellular loops. Of the seven transmembrane helices, helix 1 and 7 show higher degrees of sequence similarity than the other helices. The similarity between the extracellular domains of the chicken receptors is lower, but all have a conserved cysteine residue. In contrast, the intracellular domains (except at the C-terminus) generally have higher degrees of sequence similarity than the extracellular domains. The second intracellular domains contain a highly conserved DRYLAIV sequence.

### Chromosomal locations and syntenies

**Figure 5:**
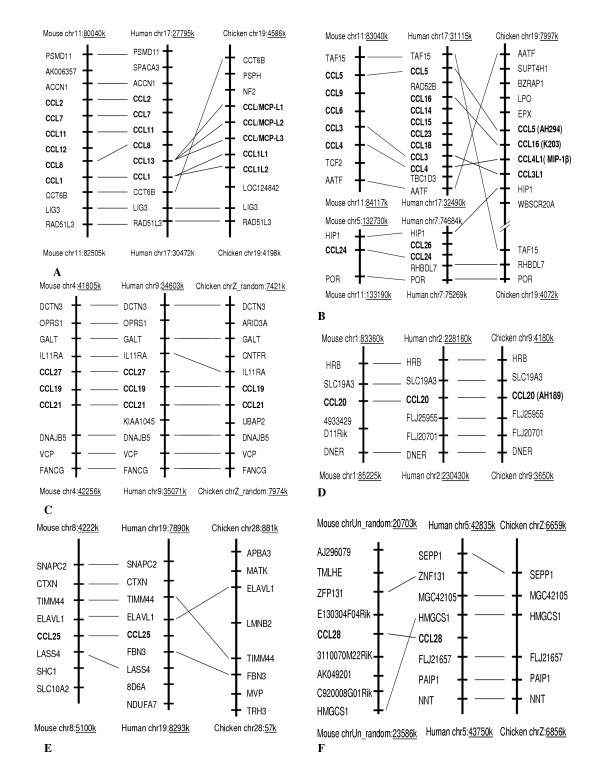
Genomic organization (syntenies) of human, mouse, and chicken CCLs, CX3CLs, and XCLs

**Figure 6:**
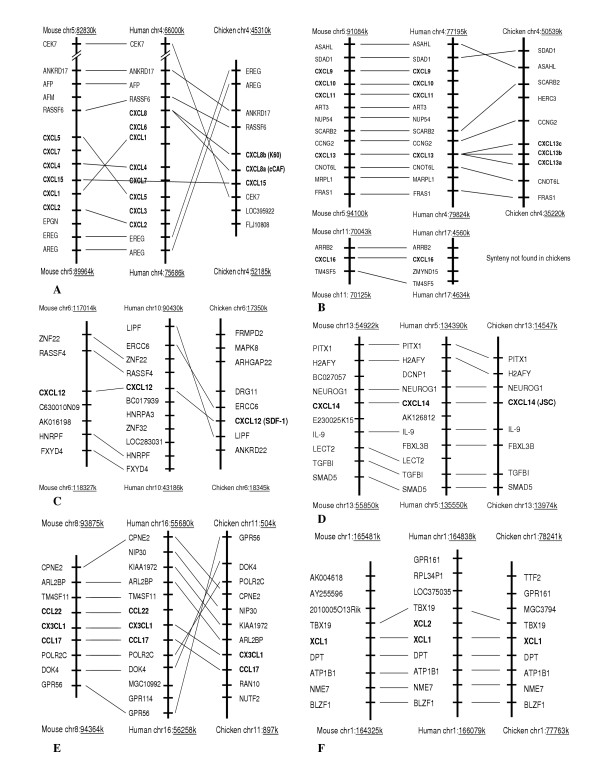
Genomic organization (syntenies) of human, mouse, and chicken CXCLs

Comparisons of the chromosomal segments containing chemokines in the human, mouse, and chicken indicate that the organization of the chemokine genes was generally conserved between chickens and mammals (Figure 5 and 6). Chicken CC and CXC chemokines are located on Chromosomes 1, 4, 6, 9, 13, 19, and Z. Like human and mouse, there are two large clusters in the chicken genome, located on Chromosome 19 and containing 9 CCL genes. Two CCL1-like (CCL1L1 and 2) and three chicken MCP-like (CCL/MCP-L1, L2, and L3) genes related to human and mouse MCPs, such as CCL2, 7, 8, 11, and 13, are in one cluster (Figure 5A), and CCL5, CCL16, CCL3L1, and CCL4L1 genes in another cluster (Figure 5B). Another CCL cluster is located on Chromosome Z containing two genes, CCL19 and CCL21 (Figure 5C). Two CXCL gene clusters are located on Chromosome 4 and contained 6 genes, two CXCL8 (CXCL8a and b) and one CXCL15 genes in one cluster (Figure 6A) and three CXCL13 (CXCL13a, b, and c) genes in another (Figure 6B). Chicken shares the syntenies with mouse and human in all these regions. There is one composite cluster containing one CX_3_CL1 and one CCL17 genes (Figure 6E). Synteny was conserved in chicken on one side of this cluster. Chicken CCL20, CXCL12, CXCL14, and XCL1 are individually located on Chromosomes 9 (Figure 5D), 6 (Figure 6C), 13 (Figure 6D), and 1 (Figure 6F), respectively, and the syntenies were highly conserved between chicken, mouse, and human in these four locations. Mammalian CCL25, CCL28, and CXCL16 were not found in the chicken genome, although the syntenies associated with CCL25 and CCL28 were also conserved in chickens. A number of human chemokines including CCL2, 7, 8, 11, 15, 18, 23, 24, and 26, CXCL1, 2, 3, 4, 5, 6, 7, 9, 10, and 11 in chemokine clusters that share syntenies with chicken clusters on Chromosome 4, and 19 were not found in the chicken genome, indicating gene duplications in mammals.

Chemokine receptor genes were also highly conserved between chicken, human, and mouse, and were similarly clustered. The largest cluster of chicken chemokine receptors was found on Chromosome 2, where 5 receptor genes (CCR2, CCR5, CCR8L1, CCR9, and XCR1) were identified. Another cluster on Chromosome 2 contains CCR4, CCR8, and CX_3_CR1 genes. Chicken CXCR1 and CXCR2 are also clustered as in mammals, but the chromosomal segment is unknown. The remaining CCR and CXCR genes are individually located on Chromosomes 3 (CCR6), 27 (CCR7), 7 (CXCR4), and 24 (CXCR5). Several human chemokine receptors, such as CCR1, CCR3, CCR10, CXCR3, and CXCR6 were not found in the chicken genome, though the syntenies associated with these receptors are present in the chicken genome.

### Gene structure

According to the chicken genome sequence, chicken chemokine genes share typical three-exon CC and four-exon CXC gene structures with mammals except for CXCL13a and CXCL13b, which have only three exons. Chicken chemokine genes are shorter than the corresponding human genes due to shorter introns in chickens. The gene structure of chemokine receptors was also conserved between chicken and mammals. The EST sequences indicate that chicken chemokine receptor genes could have up to 5 exons, though the complete sequences were not available. However, the expressed sequences show that the amino acid sequences of identified chicken receptors are mostly encoded in a single exon as are most of the mammalian chemokine receptors. Chicken ESTs aligned with the chicken genome sequence indicate that these receptor mRNAs have approximately 2 kb of 5' UTR, as do those found in humans.

### Phylogenetic analyses and nomenclatures

**Figure 7:**
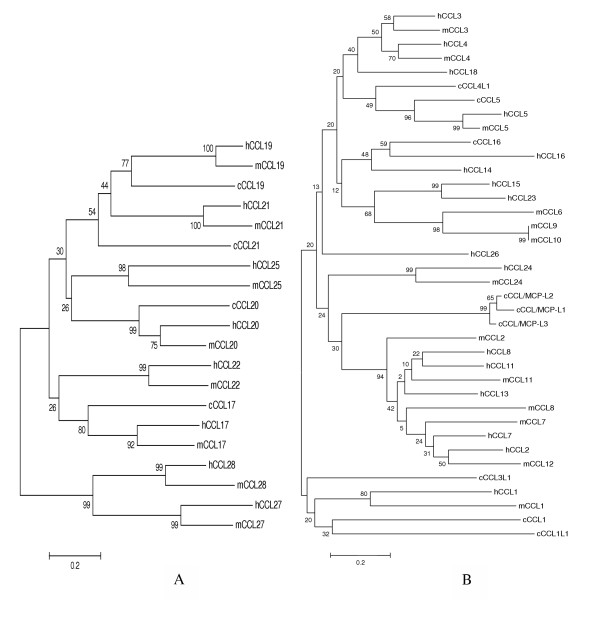
**Phylogenetic trees of the chemokine CC subfamily constructed using the amino acid sequences of chicken, human, and mouse chemokines**. The numbers on the branches are bootstrap values (percentage that the simulation supports the original interpretation). Human, mouse, and chicken are abbreviated as h, m, and c, respectively. The scale bar reflects the horizontal distance at which amino acid sequences differ by 20% between two sequences. A and B are the phylogenetic trees of CCLs that are not located on Chromosome 19 or located on Chromosome 19, respectively.

**Figure 8:**
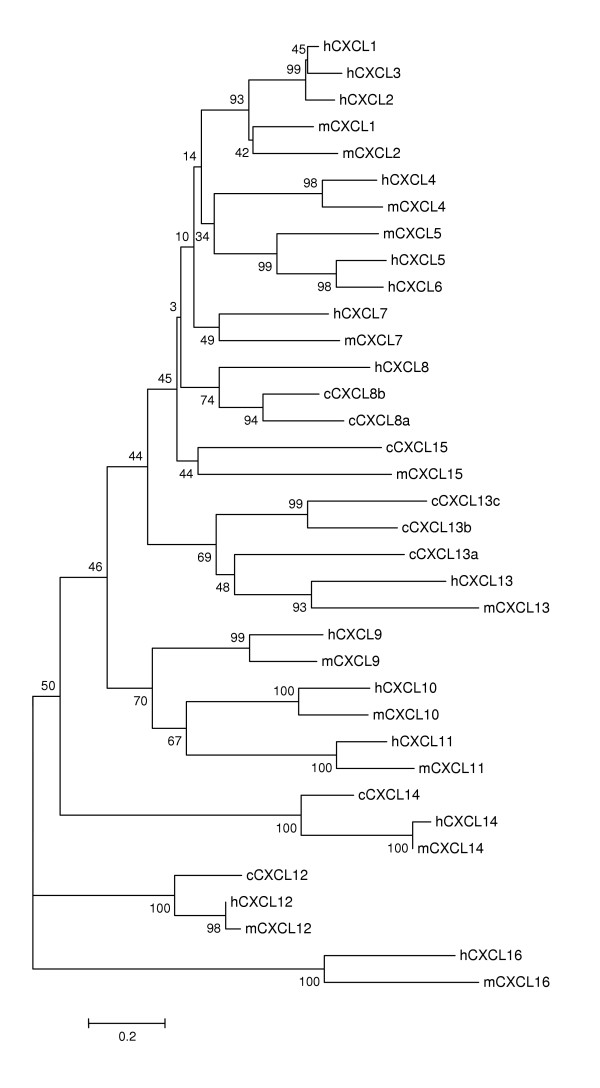
**Phylogenetic tree of the chemokine CXC subfamily constructed using the amino acid sequences of chicken, human, and mouse chemokines**. The numbers on the branches are bootstrap values (percentage that the simulation supports the original interpretation). Human, mouse, and chicken are abbreviated as h, m, and c, respectively. The scale bar reflects the horizontal distance at which amino acid sequences differ by 20% between two sequences.

The phylogenetic trees (Figure 7A,7B, and 8) show that chicken CCL5, 16, 17, 19, and 20 and all seven CXCLs are closely related to single specific human and/or mouse chemokines. The phylogenetic trees together with the syntenies associated with these genes (Figure 5 and 6) strongly indicate that these genes are the orthologs of those found in mammals; therefore, they are named accordingly. The phylogenetic results show that chickens have two CXCL8 and three CXCL13 genes (only one copy each in mammals), indicating gene duplications of these genes in aves. One chicken CXCL related to mouse CXCL15 but not to human CCLs is named as cCXCL15, which is also supported by the synteny of the chemokine cluster (Figure 6A). Chicken CCL21 is named according to relatedness to the human and mouse and the highly conserved synteny (Figure 5C). According to the phylogenetic tree in Figure 7B, two directly linked chicken CCLs are remotely related to human and mouse CCL1. The synteny associated with these genes also indicates that they may be CCL1-like genes (Figure 5A); therefore, they are named as CCL1L1 and CCL1L2. Three closely related chicken CCLs that are directly linked to the CCL1-like genes are related to a group of clustered human and mouse MCP CCLs (2, 7, 8, 11, and 13) in the phylogenetic tree. The synteny and phylogenetic tree do not provide information to a specific mammalian ortholog, though these three chicken genes are somewhat more similar to human CCL13 and mouse CCL2. The results indicate that these genes are chicken MCP-like (Figure 5A and 7B); therefore, they are named as CCL/MCP-like (CCL/MCP-L1, -L2, and -L3). A chicken CCL gene that is directly linked to CCL16 and CCL5 (Figure 5B) is distantly related to chicken CCL5 in the tree. This gene has been reported as MIP-1β-like chemokine [13], which is CCL4 in humans and mouse. Therefore, it is named as CCL4L1 in order to conform to the report. Another CCL in this cluster that does not display relatedness to other CCLs in the phylogenetic tree (Figure 7B) is named as CCL3L1 because this chemokine displays highest sequence similarity to a human CCL3-like chemokine, and it shares synteny with human CCL3 genes (Figure 5B). Overall, CXCLs are more conservative among chicken, human, and mouse than CCLs.

Chicken chemokine receptors can also be named according to mammalian nomenclature based on phylogenetic analysis (Figure 7) and syntenies. The genetic distances appear to be shorter between chicken and mammalian chemokine receptors than those between chicken and mammalian chemokines, which probably due to highly conserved transmembrane domains in these receptors. Chicken CCR4, CCR6, CCR7, CCR9, CXCR2, CXCR4, CXCR5, CX3CR1, and XCR1 are closely related to a mammalian ortholog based on the phylogenetic analysis. There are two distantly related (relative to the distance between mouse and human CCR8) CCR8 genes in chickens. The one closer to human CCR8 is named as CCR8a and the other as CCR8b. There are also two chicken CCRs closely related to human and mouse CCR2 and CCR5, but the phylogenetic analysis could not distinguish them as either CCR2 or CCR5. Because these two receptors are located in a conserved chromosomal region on chicken, human, and mouse chromosomes, these two chicken CCRs were named as CCR2 and CCR5 based on the synteny in which CCR2 is closer to XCR1 than CCR5.

In summary, 23 chemokine and 14 chemokine receptor genes were identified from the chicken genome in this study. Many chicken genes display high degrees of similarity with their human and mouse orthologs in terms of gene structure, sequence homology, and synteny. Chicken has significantly fewer CCLs, CXCLs, CCRs, and CXCRs than mammals, but it has the same number of CX_3_C, XC, and cognate receptors as mouse. The results of phylogenetic analyses generally agree with the comparative chromosomal locations and syntenies of the genes. The independent nomenclature of chicken chemokines and chemokine receptors suggests that the chicken may have ligand-receptor pairings similar to mammals. The organization of these genes suggests that there were a substantial number of these genes present before divergence between aves and mammals and more gene duplications of CC, CXC, CCR, and CXCR subfamilies in mammals than in aves after the divergence.

## Discussion

We systematically searched for chicken chemokine and chemokine receptor genes in the recently available draft chicken genome sequence. Without this information, it may have taken years to find chicken chemokines and their receptors. The independent nomenclature of chicken chemokines and chemokine receptors and mammalian chemokine-receptor binding information suggest that most of the genes have been identified. One exception was CCL25, the only known ligand of CCR9 in mammals, which was not found in this study though its receptor was identified. Likewise, CXCL14, and CXCL15 were identified in both chickens and mice, but their receptors are unknown; therefore, it is very likely that there are additional chicken chemokine and chemokine receptor genes in the chicken genome.

Although most of the systematic nomenclature of the chicken genes was unambiguous based on both phylogenetic trees and syntenies, the information that was used to name seven chicken CCLs as CCL1L1, CCL1L2, CCL3L1, CCL4L1, CCL/MCP-L1, CCL/MCP-L2, and CCL/MCP-L3 and to distinguish two chicken chemokine receptors into CCR2 and CCR5 is inadequate. CCR2 and CCR5 are closely related and tightly linked in the human, mouse, and chicken genomes. The phylogenetic analysis indicates these genes were duplicated after the divergence between mammals and aves. Chicken CCL/MCP-L1, -L2, and -L3 were related to a group of clustered mouse and human MCP CCLs; therefore, specific cognate receptors must be tested to distinguish them. In humans, the chemokines of this MCP group and MIPs, such as CCL3, CCL4, and CCL5, can bind to more than one receptor, such as CCR1, 2, 3, and/or 5, but not both CCR2 and CCR5. CCR1 and CCR3 were not found in the chicken genome and probably are not present in the species. Therefore, chicken CCR2 and CCR5 may be two receptors that recognize these two groups of CCL chemokines, such as CCR2 for MCPs and CCR5 for MIPs. Interestingly, two CCL1 like (CCL1L1 and CCL1L2) and two CCL1 receptor (CCR8a and CCR8b) genes were found in the chicken genome. The ligand-receptor binding for these four genes can not be determined in this study. Nerveless, the names assigned based on comparative analysis in this study may prove useful in order to apply the functional and physiological knowledge from other species to chickens. Further lab testing must be carried out to confirm the ligand-receptor binding and to understand their biological functions.

Chicken chemokine ESTs are highly represented in the EST database. There are several ESTs aligned to each identified chicken chemokine gene in the UCSC Genome browser. The sequences assembled from ESTs probably contained most, if not all, of the full-length chemokine mRNA sequences. Promoter sequences with a typical TATAA were detected with promoter prediction software (data not shown). However, there were only a few ESTs that partially cover chicken chemokine receptor genes. Some of these EST contain translation start sites. These EST sequences and reported complete coding sequences indicate that the amino acid sequences of chicken chemokine receptors are mostly encoded in one exon. The predicted amino acid sequences were of the expected length and aligned very well with the coding sequences of non-chicken reference genes in the UCSC genome browser. The conserved gene structure of this receptor family and high sequence similarity between chicken and mammals suggest that the predicted coding sequences were very accurate, especially for those with ESTs containing translation start sites. CCR4 is the only predicted gene that does not have a matching EST and CXCR2 is the only identified gene with partial sequence. Further study including sequencing expressed sequences is needed to confirm these genes.

## Conclusion

Based on the organization, syntenies, and phylogenetic trees of chicken, mouse, and human chemokine and chemokine receptor genes, we conclude that there may be a substantial number of chemokine and cognate receptor genes before divergence between aves and mammals. The presence of a few chicken chemokine and chemokine receptor paralogs and orthologs of the mammalian genes indicated that most chicken chemokine and the receptor genes shared common ancestors with the human and mouse genes. There were significantly more gene duplications of CC, CXC, CCR, and CXCR subfamilies in mammals than in aves after the divergence of mammals and aves. The mammalian and chicken genome sequences and the genes identified in this study can be used for further investigation of the molecular evolution of these gene families and as a model for the study of the divergence between aves and mammals. Avian and mammalian species may share similar chemokine-receptor binding patterns. The results of this study may be used as functional inferences for these chicken genes before they are experimentally tested.

## Methods

### Gene identification

To identify syntenies, genes closely linked to human and mouse chemokines were identified and localized on the chicken genome using the UCSC genome browser [25]. Expressed Sequence Tags (ESTs) and chicken mRNA sequences in the corresponding chromosomal regions were then identified and, if necessary, assembled with the CAP3 program [26,27]. These sequences were aligned with the corresponding chicken genomic sequence and any deletions or insertions corrected. Sequences were then submitted to ORF Finder (Open Reading Frame Finder) [28] and the open reading frames were used as queries in BLASTP [29,30] searches against the non-redundant protein database in Genbank [31]. Sequences that produced significant alignments with chemokines were identified as putative chicken chemokine sequences. To identify chicken chemokine receptors, all sequences of putative chicken chemokine receptors including ESTs, mRNAs, and predicted sequences were retrieved from the UCSC Genome Browser. The identified ESTs were used to determine the translation start sites for the receptors. If the translation start sites could not be determined from ESTs, translation start sites were based on the most likely predicted sequences from non-chicken reference genes in the UCSC Genome Browser.

### Sequence analyses

Complete amino acid sequences of currently known human and mouse chemokines were retrieved from Genbank. The amino acid sequences of all putative chicken chemokines were predicted based on the open reading frames of the expressed nucleotide sequences (ESTs or mRNAs). The amino acid sequences were grouped according to CC, CXC, and CX_3_C motifs and aligned using the ClustalW program [32,33]. The similarity of the amino acid sequences was determined based on alignments with the most likely human or mouse orthologs. Human CCR1 was included in the multiple alignments of chicken chemokine receptors for comparison. The seven transmembrane domains were predicted using the SMART program [34].

For comparison, human chemokines hCCL1 (GenBank accession number: (NM_002981), hCCL2 (BC009716), hCCL3 (BC071834), hCCL4 (NM_002984), hCCL5 (BC008600), hCCL7 (NM_006273), hCCL8 (NM_005623), hCCL11 (BC017850), hCCL13 (BC008621), hCCL14(BC045165), hCCL15 (NM_032964), hCCL16 (NM_004590), hCCL17 (BC069107), hCCL18 (BC069700), hCCL19 (BC027968), hCCL20 (BC020698), hCCL21 (BC027918), hCCL22 (BC027952), hCCL23 (NM_145898), hCCL24 (BC069072), hCCL25 (NM_005624), hCCL26 (BC069394), hCCL27 (AJ243542), hCCL28 (AF220210), hCXCL1 (BC011976), hCXCL2 (BC015753), hCXCL3 (BC065743), hCXCL4 (NM_002619), hCXCL5 (BC008376), hCXCL6 (BC013744), hCXCL7 (BC028217), hCXCL8 (BC013615), hCXCL9 (BC063122), hCXCL10 (BC010954), hCXCL11 (BC012532), hCXCL12 (BC039893), hCXCL13 (BC012589), hCXCL14 (BC003513), and hCXCL16 (BC017588), and hCX_3_CL1(NM_002996) and mouse chemokines CCL1 (NM_011329), mCCL2 (NM_011333), mCCL3 (NM_011337), mCCL4 (NM_013652), mCCL5 (BC033508), mCCL6 (BC002073), mCCL7 (BC061126), mCCL8 (NM_021443), mCCL9 (NM_011338), mCCL10 (U15209), mCCL11 (NM_011330), mCCL12 (BC027520), mCCL17 (BC028505), mCCL19 (BC051472), mCCL20 (BC028504 (NM_009138), mCCL27 (BC028511), mCCL28 (BC055864), and mCX_3_CL1 (BC054838) were retrieved from the GenBank. Reported chicken chemokines K60 (Y14971), cCAF (M16199), MIP-1β (AJ243034), k203 (Y18692), AH294 (AY037859), AH221 (AY037860), AH189 (AY037861), JSC (AF285876), SDF-1(BX936268), Clone 391 (L34552) and lymphotactin (AF006742) are included in this study. Rat (BC070938) and monkey (AF449286) CX_3_CL1 were also retrieved for CX_3_CL sequence analysis. There are several human chemokine-like genes in the human genome, which were not included in this study.

Human and mouse chemokine receptors hCCR1 (NM_001295), hCCR2 (NM_000647), hCCR3 (NM_001837), hCCR4 (NM_005508), hCCR5 (NM_000579), hCCR6 (NM_004367), hCCR7 (NM_001838), hCCR8 (NM_005201), hCCR9 (NM_006641), hCCR10 (AY429103), hCXCR1 (NM_000634), hCXCR2 (BC037961), hCXCR3 (NM_001504), hCXCR4 (AY728138), hCXCR5 (NM_032966), hCXCR6 (NM_006564), hCX3CR1 (NP_001328), and hXCR1 (NM_005283), mCXCR1 (AY749637), mCXCR2 (NM_009909), mCXCR3 (NM_009910), mCXCR4 (NM_009911), mCXCR5 (NM_007551), mCXCR6 (NM_030712), mCCR1 (NM_009912), mCCR2 (NM_009915), mCCR3 (NM_009914), mCCR4 (NM_009916), mCCR5 (NM_009917), mCCR6 (NM_009835), mCCR7 (NM_007719), mCCR8 (NM_007720), mCCR9 (NM_0099130), mCCR10 (AF215982), mCX3CR1 (NM_009987), and mXCR1 (NM_011798), and reported chicken cCCR2 (CAF28776), cCCR5 (BI393893, CAF28777), cCCR8L1(CAF28778), cCCR9 (CAF28781), cCXCR1 (AAG33964), cCXCR4 (NP_989948), and cXCR1 (CAF28779), were also retrieved from GenBank for comparisons.

Phylogenetic analyses of protein sequences of chicken, human, and mouse chemokines and chemokine receptors were based on the amino acid sequences using neighbor-joining with options selected for bootstrap test, pairwise deletion and Poisson correction, using MEGA3 [35,36]. For ligand-receptor inference, the first 20 amino acids (leading peptide) of all chemokines were removed before the phylogenetic analysis and chicken CCLs were divided into two groups, one group located on Chromosomes 4 and 19 and the other from other chromosomes. Syntenies, phylogenetic trees, and sequence homologies were the combined information used for naming chicken chemokines and their cognate receptors according to the recommendations of the IUIS/WHO Subcommittee on Chemokine Nomenclature [37]. These chicken genes were named according to their closest predicted human or mouse orthologs if all information supports the nomenclature. If there was more than one chicken gene similar to a human and/or mouse gene, these gene was named as in the human and/or mouse followed by a letter with alphabet order. If a specific human or mouse ortholog could not be reliablely determined, the chicken genes were named according to a closest human or mouse ortholog followed by an "L" and a number based on the information available. This nomenclature also used the existing systematic names reported in the literature to avoid confusion.

### Polymerase chain reaction (PCR) and DNA sequencing

Chicken EST or mRNA sequences were identified for all chemokine genes. All sequences contained complete putative open reading frames except for CX_3_CL1. However, partial chicken CX_3_CL1 gene sequences (BM426140, BI066258, and CR389767) were identified, with a gap of 123 nucleotides between the ESTs. Forward (TGTGACATCGGGAGTCGCTAC) and reverse (AAAATCCCCAGCGTTTGCTACT) PCR primers were used to amplify across the gap using cDNA prepared from white blood cells. PCR was performed as follows: An initial denaturation step at 94°C for 2 min and 35 cycles of denaturation, annealing, and extension at 94°C for 30 sec, 59°C for 45 sec, and 72°C for 1 min., and a final extension step was carried out at 72°C for 10 min. Unincorporated nucleotides were removed from amplified PCR products using BioMax spin-50 mini-columns (Millipore, Billerica, MA). BigDye terminator cycle sequencing reaction kits and an ABI Prism 377XL DNA Sequencer (Applied Biosystems) were used for DNA sequencing.

## List of abbreviations

Abbreviations: cCAF, chicken chemotactic and angiogenic factor; JSC, Jun-suppressed chemokine; SDF-1, stromal cell-derived factor-1; MCPs, monocyte chemoattractant proteins; MIPs, macrophage inflammatory proteins.

## Authors' contributions

JW collected most of the data and drafted the manuscript. DLA contributed to the interpretation of the data and final approval of the manuscript. AY performed the DNA sequencing and assisted with the preparation of the manuscript. SHS and YJ designed computer programs to search chicken chemokine sequences in chicken EST database. JJZ provided the conception and design of the study, collected some of the data, conducted phylogenetic analysis, and revised the manuscript.

**Figure 9:**
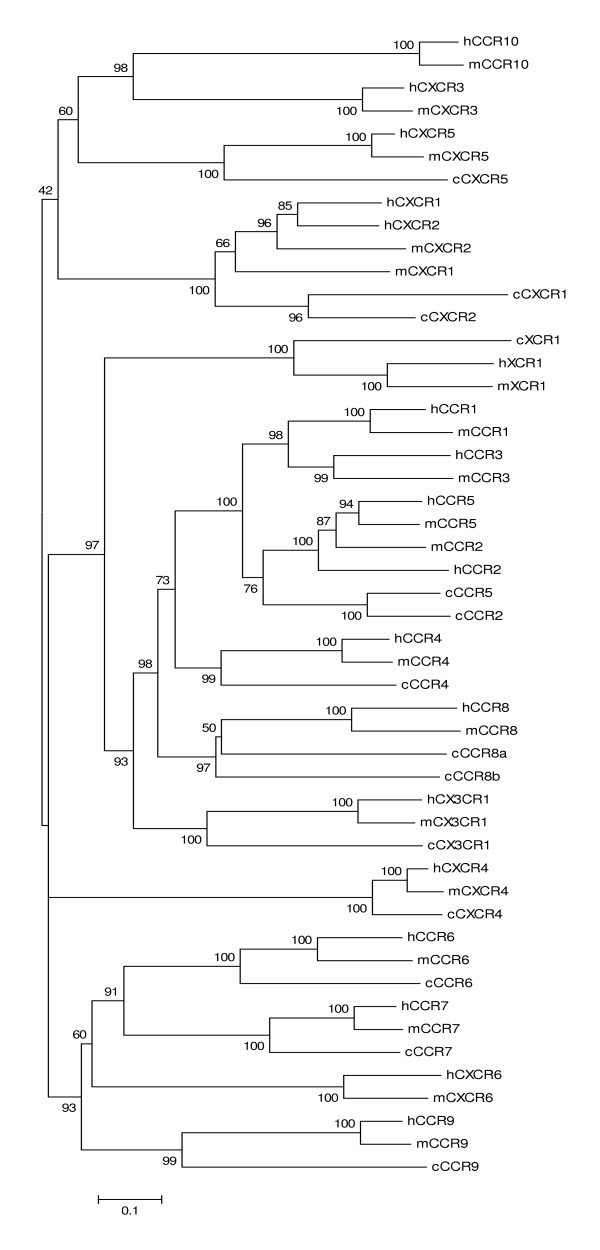
**Phylogenetic tree of chemokine receptors constructed using the amino acid sequences of chicken, human, and mouse chemokine receptors**. The numbers on the branches are bootstrap values (percentage that the simulation supports the original interpretation). Human, mouse, and chicken chemokines are abbreviated as h, m, and c, respectively, followed by the receptor named. The scale bar reflects the horizontal distance at which amino acid sequences differ by 10% between two sequences.

**Table 2:** Systematic names, chromosomal locations (kb), and putative identified cognate receptors of chicken chemokines^1^

Nomenclature	Chromosomal location	Ligand name	Putative receptor	Chromosomal location
CCL1L1	chr19:4,491–4,492	I-309/TCA	CCR8a and/or CCR8b	chr2:43,465–43,469
CCL1L2	chr19:4,493–4,495			chr2:41,804–41,811
CCL/MCP-L1	chr19:4,495–4,496	MCP-?		
CCL/MCP-L2	chr19:4,498–4,499	MCP-?	CCR2	chr2:41,768–41,769
CCL/MCP-L3	chr19:4,507–4,508	MCP-?		
CCL16	chr19:258–261	HCC		
CCL3L1	chr19:240–242	MIP-1α		
CCL4L1	chr19:250–253	MIP-1β	CCR5	chr2:41,784–41,786
CCL5	chr19:263–266	RANTES		
CCL17	chr11:768–771	TARC	CCR4	chr2:43,501–43,503
CCL19	chrZ_random:7,804–7,809	MIP-3β	CCR7	chr27_random:661–673
CCL21	chrZ_random:7,810–7,810	SLC		
CCL20	chr9:4,119–4,122	MIP-3α	CCR6	chr3:38,589–38,596
Not found^2^		CCL25	CCR9	chr2:41,880–41,882
CXCL8b	chr4:51,462–51,466	IL-8	CXCR2	chrUn:136,108–136,109
CXCL8a	chr4:51,475–51,479		CXCR1^3^	chrUn:25,460–25,462
CXCL12	chr6:18,184–18,195	SDF-1	CXCR4	chr7:31,441–31,443
CXCL13a	chr4:35,453–35,455	BCA-?		
CXCL13b	chr4:35,455–35,457	BCA-?	CXCR5	chr24:5,242–5,247
CXCL13c	chr4:35,457–35,459	BCA-?		
CXCL14	chr13:14,231–14,239	BRAK	Unknown^4^	
CXCL15	chr4:51,500–51,501	Lungkine	Unknown^4^	
CX_3_CL1	chr11:758–764	Fractalkine	CX_3_CR1	chr2:43,480–43,490
XCL1	chr1:780,81–78,086	Lymphotactin	XCR1	chr2:41,831–41,833

^1 ^The systematic naming, ligand naming, and putative receptors are according to [37] and [38].

^2 ^CCL25 was not identified in this study.

^3 ^The ligand and receptor binding has been experimentally tested [39]

^4 ^The information is currently not available in humans or mice.
